# Effects of Telehealth-Supervised Respiratory Exercise Training on Respiratory Function, Fatigue, Quality of Life, and Functional Capacity of Patients with Multiple Sclerosis

**DOI:** 10.3390/medicina61040651

**Published:** 2025-04-02

**Authors:** Şeyda Öznur Ayçiçek, Abdulkadir Tunç, Cahit Bağcı

**Affiliations:** 1 Department of Physiology, Faculty of Medicine, Sakarya University, Sakarya 54050, Turkey; seydaaycicek@subu.edu.tr (Ş.Ö.A.);; 2 Department of Physiotherapy and Rehabilitation, Faculty of Health Sciences, Sakarya University of Applied Sciences, Sakarya 54050, Turkey; 3 Department of Neurology, Faculty of Medicine, Sakarya University, Sakarya 54050, Turkey

**Keywords:** telerehabilitation, multiple sclerosis, respiratory function tests, fatigue, quality of life

## Abstract

*Background and Objectives*: Telerehabilitation (TR) offers an innovative approach to overcome accessibility challenges in managing multiple sclerosis (MS). This exploratory study evaluated the efficacy of integrating respiratory exercises into TR programs for improving respiratory function, fatigue, and quality of life. *Materials and Methods*: A randomized controlled trial involving 48 MS patients randomized into TR and control groups was conducted. Both groups performed respiratory exercises over eight weeks. Pulmonary function, fatigue severity (FSS), quality of life (MSQOL-54), and functional capacity (6MWT) were assessed before and after the intervention. *Results*: Both groups demonstrated significant within-group improvements in FEV1 (L), PEF (L), FEF%25–75 (L), FSS, MSQOL-54 physical and mental subscales, and the 6MWT distance (*p* < 0.05). The TR group exhibited unique improvements in FEV1 (%) and slightly greater reductions in fatigue, although the intergroup differences were not statistically significant. *Conclusions*: Telerehabilitation incorporating respiratory exercises effectively enhances the respiratory function, fatigue, and quality of life of MS patients, suggesting a viable alternative to conventional rehabilitation. Future studies should focus on advanced-stage MS, long-term sustainability, and technological integration to optimize the potential of TR.

## 1. Introduction

Multiple sclerosis (MS) is a chronic autoimmune disease of the central nervous system characterized by demyelination and axonal damage that can impair motor, sensory, and autonomic functions [1]. Global estimates suggest that over 2.8 million individuals, predominantly women of working age, are affected by MS [2,3]. Among its many manifestations, MS often results in a wide array of symptoms, including fatigue, motor impairments, and respiratory dysfunction, particularly in the progressive stages of the disease [4]. Respiratory complications are of particular concern, as studies have shown that weakened respiratory muscles and a reduced lung capacity are prevalent in individuals with MS [5]. These impairments not only compromise quality of life but also increase the risk of secondary complications, such as respiratory infections, and reduce functional independence [6]. In addition to the challenges posed by disease progression, medications prescribed for regulatory and protective purposes may inadvertently affect respiratory functions [7]. Therefore, developing strategies that focus on maintaining respiratory muscle strength, optimizing lung capacity through effective breathing techniques, and improving functional capacity to prevent further complications in patients with advanced stages of MS is imperative [8,9]. Rehabilitation programs incorporating respiratory exercises have demonstrated the potential to mitigate these challenges by improving respiratory function, alleviating fatigue, and enhancing overall quality of life [10].

Telerehabilitation (TR) has emerged as a transformative telemedicine approach that addresses the physical and logistical barriers often faced by MS patients. This method leverages telecommunication technologies to deliver rehabilitation services remotely, enabling patients to receive therapy in the comfort of their homes while maintaining real-time interactions with healthcare providers [11]. Telerehabilitation is particularly advantageous for individuals living in remote areas or those with mobility limitations, as it eliminates the need for frequent in-person visits to healthcare facilities [12]. Research has increasingly highlighted the efficacy of TR in improving patient outcomes across a variety of conditions, including neurological diseases, stroke, and pulmonary rehabilitation [13,14,15,16]. For MS, TR has demonstrated the potential to deliver personalized therapeutic interventions that address both physical and cognitive challenges. Furthermore, advancements in synchronous (live video consultations) and asynchronous (mobile applications and prerecorded videos) telerehabilitation methods have enhanced patient engagement, adherence, and satisfaction [17,18]. Wearable devices and integrated sensors have further expanded the possibilities for continuous monitoring and personalized care, ensuring that therapy is tailored to individual needs [19].

This study aims to evaluate the feasibility and effectiveness of integrating respiratory exercises into TR programs for MS patients, with a specific focus on alleviating fatigue and improving pulmonary function, functional capacity, and quality of life.

## 2. Materials and Methods

### 2.1. Study Design and Participants

**Figure 1:**
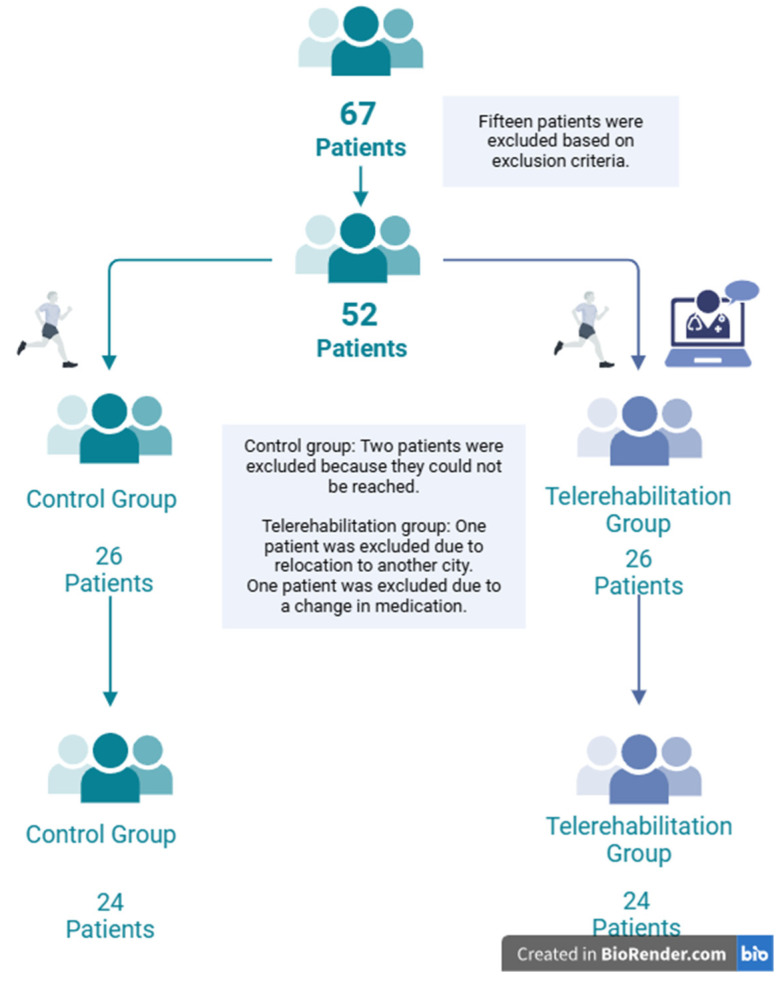
Flowchart of patient selection and group allocation. The figure illustrates the process of patient selection, exclusion, and final allocation into the study groups.

This exploratory randomized controlled trial was conducted to evaluate the effectiveness of respiratory exercises delivered remotely under supervision (TR group) compared to an identical respiratory exercise program performed independently at home without supervision (control group) in patients with MS. The study took place at the neurology outpatient clinics of Sakarya Training and Research Hospital between August 2022 and March 2023, following ethical approval from Sakarya University’s Clinical Research Ethics Committee (approval number: 23.06.2022-82). All participants provided written informed consent in accordance with the Declaration of Helsinki. The inclusion criteria were a diagnosis of MS according to the 2017 McDonald criteria [1], an age between 18 and 65 years, an expanded disability status scale (EDSS) score of 0–7, and the ability to provide informed consent and participate in the study. Patients with comorbidities affecting respiratory capacity (e.g., chronic obstructive pulmonary disease or asthma), recent medication changes, corticosteroid use, MS relapses within the past three months, significant visual impairments, pregnancy, or conditions preventing a proper assessment were excluded. The participants had no prior exposure to structured respiratory physiotherapy programs or formal educational sessions on respiratory training to ensure that baseline respiratory function was not influenced by previous interventions. Initially, 67 patients diagnosed with MS were screened for eligibility, of whom 15 did not meet the inclusion criteria. Thus, 52 patients were randomized into two groups: the TR group (*n* = 26) and the control group (*n* = 26). During the study, 4 participants (2 from each group) dropped out. The final analysis therefore included 24 participants in each group. The sample size was determined based on an a priori power analysis using GPower (version 3.1.9.4), which indicated that a total of 48 participants (24 per group) were sufficient to achieve 95% statistical power, an alpha level of 0.05, and an estimated effect size of 0.95. Considering possible dropouts, a slightly higher initial sample size was planned (Figure 1). Baseline demographic and clinical data (e.g., age, sex, BMI, EDSS score, smoking status, last relapse date, and MS type) were collected.

### 2.2. Intervention

The participants were randomized into two groups (TR and control) via a computer-generated sequence and were instructed to avoid other exercise programs during the study. The primary intervention consisted exclusively of respiratory exercise training delivered remotely via telehealth. Participants in the telerehabilitation group (TR group) performed structured respiratory exercises under the supervision of a researcher through real-time video calls twice weekly over an 8-week period, with sessions providing real-time feedback and a progressive adjustment of exercise intensity. In contrast, participants in the control group (CG) received written instructions for the identical respiratory exercises and performed them independently at home without supervision or real-time feedback. All participants attended an initial in-person session for instruction and demonstration of respiratory muscle training to ensure proper technique and comprehension of the exercise protocol [8,17,20,21].

### 2.3. Outcome Measures

**Figure 2:**
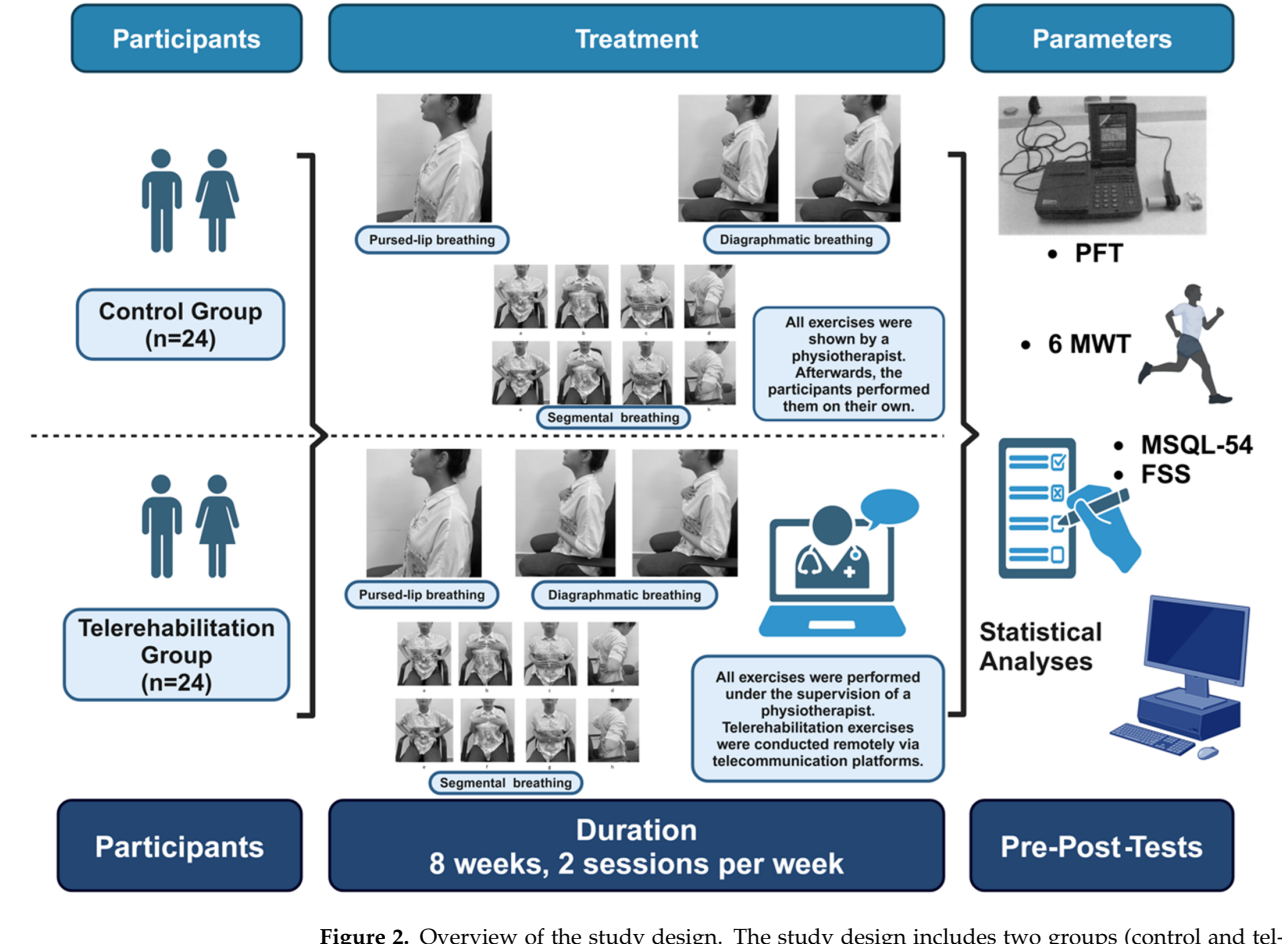
Overview of the study design. The study design includes two groups (control and telerehabilitation, *n* = 24 each), with both undergoing breathing exercises (pursed-lip, diaphragmatic, and segmental). Parameters assessed: pulmonary function tests (PFTs), 6 min walk test (6MWT), Multiple Sclerosis Quality of Life-54 (MSQL-54), and Fatigue Severity Scale (FSS). The telerehabilitation group performed exercises remotely under a physiotherapist’s supervision. Duration: 8 weeks, 2 sessions/week.

The outcomes were assessed at baseline (preintervention) and at the end of the 8-week intervention period by the same researcher (Figure 2).

Respiratory function was assessed via a spirometry test (MIR Spirolab III), a reliable and widely used device for evaluating pulmonary function. The participants were provided with detailed instructions on the testing procedure to ensure consistency and accuracy. They were advised to avoid any activities or substances that could influence respiratory performance, such as eating, consuming caffeine, or engaging in strenuous physical activity, for at least two hours before the test. The test was conducted in a seated position to minimize postural effects on lung capacity. Each participant was fitted with a nose clip to prevent air escape and was instructed to use a disposable mouthpiece to maintain hygiene. They were guided to perform a series of breathing maneuvers per standardized spirometry protocols, ensuring adequate practice trials before recording the measurements. The following parameters were evaluated: forced vital capacity (FVC), the total volume of air exhaled forcefully after deep inhalation, reflects the overall capacity of the lungs; forced expiratory volume in 1 s (FEV1), the volume of air expelled during the first second of forced exhalation, is an indicator of airflow limitation; the FEV1/FVC ratio, the proportion of FVC exhaled in the first second, is used to identify obstructive airway conditions; peak expiratory flow (PEF), the maximum speed of airflow achieved during forced expiration, represents the peak force of respiratory muscles; and forced expiratory flow (FEF25–75), the average airflow rate during the middle half of exhalation, is a sensitive measure of small airway function. To ensure reproducibility, each test was performed three times, and the highest values meeting the quality control standards were recorded. The procedure adhered to the American Thoracic Society/European Respiratory Society (ATS/ERS) guidelines to maintain consistency and reliability across participants [22].

Fatigue was measured via the Fatigue Severity Scale (FSS), a 9-item self-report questionnaire validated for MS populations, where higher scores indicate greater fatigue [23]. Quality of life was assessed via the Multiple Sclerosis Quality of Life-54 [MSQOL-54), which provides mental and physical composite scores on the basis of patient-reported data [24]. Functional capacity was evaluated through the 6 min walk test (6MWT), which was conducted along a 30 m corridor, with the distance walked, blood pressure, heart rate, and oxygen saturation recorded before and after the test [25].

### 2.4. Respiratory Exercise Program

The breathing exercises included diaphragmatic breathing, pursed-lip breathing, and inspiratory muscle training techniques. These exercises were selected to enhance respiratory muscle performance, optimize ventilatory efficiency, and reduce dyspnea during physical activity. Diaphragmatic breathing was included to improve breathing control and coordination rather than to strengthen the diaphragm itself. Additionally, pursed-lip breathing and inspiratory muscle training were incorporated to promote lung volume expansion and increase inspiratory muscle endurance. Pursed-lip breathing requires partic- ipants to inhale deeply through their nose and exhale slowly through pursed lips, a tech- nique aimed at prolonging exhalation, reducing air trapping, enhancing alveolar ventilation, and minimizing the work of breathing. This exercise was encouraged during rest and low-intensity physical activity to help participants manage dyspnea [26]. Diaphragmatic breathing, performed in a seated position, focuses on abdominal expansion during inhalation and contraction during exhalation while exhaling through pursed lips. The participants monitored their abdominal movement by placing one hand on the abdomen and the other on the chest to ensure minimal accessory muscle use, thereby improving diaphragmatic strength, increasing lung ventilation, and alleviating fatigue. Finally, segmental breathing exercises targeted specific regions of the chest wall to enhance localized lung ventilation, increase alveolar oxygenation, and improve chest mobility. This technique involves tactile stimulation applied to areas such as the lateral, upper, lower, and postero- basal chest regions to guide participants in expanding the targeted area during inspiration and exhaling slowly through pursed lips. Resistance was progressively introduced as participants advanced, further strengthening inspiratory muscles and optimizing lung function [27].

### 2.5. Statistical Analysis

The data were analyzed via SPSS version 25.0. Normality was assessed with the Shapiro–Wilk test. For normally distributed data, independent samples were analyzed with the independent *t*-test, and paired samples were analyzed with the paired *t*-test. Nonnormally distributed data were analyzed via the Mann–Whitney U test for independent samples and the Wilcoxon test for paired samples. Relationships between categorical variables were evaluated via the chi-square test. A *p*-value of ≤0.05 was considered statistically significant.

## 3. Results

### 3.1. Baseline Characteristics of Patients

The study initially included 26 patients in each group; however, owing to relocation (*n* = 3) and medication changes (*n* = 1), the final sample consisted of 24 patients in both the TR and control groups. Baseline sociodemographic and clinical characteristics, including age, BMI, EDSS score, sex distribution, and smoking status, were comparable between the groups (all *p* > 0.05, Table 1).

### 3.2. Pretreatment Functional and Pulmonary Assessments

Pretreatment comparisons revealed no significant differences between the groups in pulmonary function test (PFT) outcomes, including FVC, FEV1, PEF, and FEF%25–75 values, expressed both in liters and percentage of predicted values (all *p* > 0.05, Supplementary Table S1). Additionally, functional assessments encompassing the FSS, the physical and mental subscale scores of the MSQL-54, and the 6MWT outcomes similarly demonstrated no significant baseline differences between the groups (all *p* > 0.05, Supplementary Table S2).

### 3.3. Pulmonary Function Test Outcomes Posttreatment

The within-group analysis of PFT outcomes revealed significant improvements in several parameters. In the TR group, the FEV1 (L), PEF (L), PEF (%), and FEF%25–75 (L) significantly increased after treatment (*p* < 0.05), whereas the FEV1 (%) improved exclusively in this group (*p* < 0.05). The control group demonstrated similar improvements in FEV1 (L), PEF (L), PEF (%), and FEF%25–75 (L), although the magnitude of changes was not significantly different from that of the TR group (*p* > 0.05 for intergroup comparisons, Table 2). These findings highlight the effectiveness of both interventions at enhancing respiratory function but do not suggest a superior benefit of TR.

### 3.4. Functional and Quality-of-Life Outcomes

Posttreatment assessments indicated significant within-group improvements across multiple domains for both groups. FSS scores, MSQL-54 physical component scores (PCSs), mental component scores (MCSs), heart rate changes during the 6MWT, and total walking distance all demonstrated significant gains (*p* < 0.05, Table 3). Despite these improvements, intergroup comparisons yielded no statistically significant differences across these parameters (all *p* > 0.05). The oxygen saturation levels during the 6MWT remained stable before and after treatment in both groups, with no meaningful intra- or intergroup differences (*p* > 0.05).

## 4. Discussion

This study evaluated the effects of a TR program incorporating respiratory exercises on MS patients. Both the telehealth-supervised respiratory exercise group (TR group) and the unsupervised respiratory exercise control group showed significant within-group improvements in pulmonary function, fatigue levels, functional capacity, and quality of life. However, no statistically significant differences in any outcome measure were observed between the two groups. These findings suggest that both structured TR and home-based respiratory training can lead to improvements in MS-related respiratory and functional outcomes.

Respiratory exercise significantly enhanced pulmonary function in both groups, with notable improvements in FEV1 (L), PEF (L), and FEF%25–75 (L). These findings align with those of previous studies demonstrating the benefits of structured respiratory training in MS patients, particularly in improving pulmonary function and ventilatory efficiency. Recent studies, such as Nazem et al. [21], have highlighted the role of breathing exercises in enhancing muscle activity and respiratory function in MS patients, reinforcing their importance beyond general fitness. Similarly, Jallouli et al. [28] reported that self-paced training programs incorporating breathing techniques led to significant improvements in lung function and cardiopulmonary fitness, further supporting the efficacy of structured respiratory rehabilitation strategies for patients with MS and other chronic conditions. Consistent with prior research, both TR- and home-based respiratory training in our study led to significant improvements, reinforcing the effectiveness of structured home-based rehabilitation programs. However, a distinctive increase in FEV1 (%) was observed in the TR group, which may indicate a potential advantage of supervised remote interventions in reinforcing proper breathing mechanics, optimizing adherence, and ensuring progressive intensity adjustments. Unlike studies focused on inspiratory muscle training, which is primarily aimed at increasing inspiratory muscle strength, our intervention emphasized ventilatory efficiency, breathing coordination, and respiratory endurance rather than isolated muscle strengthening [29,30,31]. While this suggests that TR may provide added benefits beyond home-based training alone, this finding should be interpreted with caution, given the study’s sample size and the absence of long-term follow-up data. Future studies should explore whether the observed improvements in FEV1 (%) are sustained over time and whether the advantages of TR extend to long-term respiratory function preservation in MS patients. Additionally, recent research on long COVID has similarly demonstrated that remote pulmonary rehabilitation enhances lung function and respiratory endurance [20], reinforcing the potential of TR as a scalable and accessible rehabilitation tool. These findings highlight the potential for hybrid intervention models that combine telemonitoring, wearable technology, and structured self-management strategies to optimize long term respiratory rehabilitation outcomes in patients with MS and other chronic conditions. Both groups experienced significant reductions in fatigue severity, as indicated by decreased FSS scores. Fatigue is a debilitating symptom for MS patients, and its management is critical for improving quality of life. Prior studies, such as Ray et al. [30], have emphasized the role of respiratory exercises in alleviating fatigue by enhancing respiratory muscle strength and endurance, which parallels the reductions in fatigue observed in our study. Although no significant intergroup differences were noted, the slightly greater fatigue reduction in the TR group may be attributed to real-time supervision optimizing exercise engagement, the psychological benefits of remote support, and the reduced logistical burden allowing for better energy allocation. These findings align with prior studies demonstrating the role of structured, remotely supervised interventions in mitigating fatigue in MS patients [8,30,32].

Jeong et al. [32] reported improved quality of life across all subscales of the MSQOL-54 with TR, aligning with the positive trends we observed in the PCS and MCS subscales. These improvements, coupled with enhanced 6MWT performance, reflect the positive impacts of TR on both quality of life and functional outcomes. Additionally, the increased walking distance observed in the 6MWT further supports the role of TR in improving physical endurance and functional mobility in MS patients. The observed improvements may be attributed to enhanced respiratory efficiency, structured progression, and increased adherence to training sessions, reinforcing the capacity of TR to facilitate endurance gains. These findings align with those of prior research demonstrating the benefits of TR in promoting physical activity and gait function [8,33]. While this study did not assess maximum inspiratory and expiratory pressures (MIP/MEP), future research could integrate these measures to quantify the role of respiratory muscle strength in endurance improvements. Given that respiratory dysfunction is a major contributor to fatigue in patients with MS and other chronic conditions, assessing MIP/MEP could provide valuable insights into the physiological mechanisms underlying endurance gains and fatigue alleviation in TR-based interventions [20].

The comparable outcomes between TR and conventional unsupervised rehabilitation suggest that TR is a viable alternative to home-based training. The absence of significant intergroup differences may be due to the structured nature of the control group’s program, high patient adherence, and the inherent benefits of respiratory exercises, which independently improve pulmonary function and fatigue [31,34]. This aligns with findings from Fjeldstad-Pardo et al. [33], who reported no significant differences in gait or balance outcomes between TR and in-person physical therapy, reinforcing the effectiveness of structured home-based rehabilitation. Research indicates that intrinsic patient motivation and engagement play crucial roles in rehabilitation outcomes, potentially mitigating the impact of supervision differences [20,35]. Beyond its demonstrated efficacy, TR offers additional advantages by addressing logistical barriers, minimizing travel-related constraints, and ensuring continuous professional supervision from home. These benefits make it particularly valuable for individuals with mobility challenges or those living in remote areas [8,17,20]. For individuals with MS, who often face difficulties accessing specialized rehabilitation services due to disability or geographic distance, TR serves as a vital bridge to care. This aligns with findings from Yeroushalmi et al. [17], who demonstrated that telemedicine interventions, including TR, effectively close accessibility gaps, reduce costs, and yield high satisfaction rates among both patients and providers. Additionally, their review underscores the feasibility of remote neurological assessments and long-term rehabilitation management, reinforcing the role of TR in delivering scalable, patient-centered solutions. Moreover, TR enhances patient adherence through structured feedback and engagement while alleviating the psychological stress associated with in-person visits.

These advantages position TR as a highly adaptable, patient-centered rehabilitation model, particularly for managing chronic conditions such as MS. However, as emphasized in prior studies, standardizing TR protocols remains essential for optimizing treatment efficacy and ensuring consistency in the intervention frequency, duration, and progression [35,36]. The logistical advantages of TR, such as reduced travel demands and continuous remote supervision, enhance patient adherence and engagement, reinforcing its role as a scalable rehabilitation strategy. Integrating emerging technologies, including wearable devices and telemonitoring tools, could further refine TR by enabling real-time tracking of respiratory function, physical activity, and fatigue levels. These innovations provide clinicians with dynamic feedback, allowing for more personalized and adaptive rehabilitation interventions [8,17]. Future research should explore the long-term sustainability of these technologies, assessing their impacts on patient adherence, rehabilitation outcomes, and disease progression of MS. This research aligns with broader trends in digital health, where telehealth innovations are increasingly recognized for improving accessibility and personalized care for chronic conditions [8,17]. Additionally, a more detailed evaluation of specific respiratory metrics, such as inspiratory and expiratory pressures, may offer deeper insights into the physiological benefits of TR, further optimizing its effectiveness.

A key limitation of this study is its small sample size, which, coupled with the number of multiple comparisons, increases the risk of type I error. Moreover, the single-center design may limit the generalizability of the results to the broader MS population. Notably, a formal power analysis was not conducted; rather, recruitment was based on feasibility considerations and prior research on telerehabilitation for MS. Additionally, participants were predominantly in the early to moderate stages of MS, meaning that the findings may not fully apply to patients with advanced disease who may require more intensive rehabilitation support. The absence of long-term follow-up prevents conclusions regarding the sustainability of TR benefits over time, including adherence trends and potential effects on disease progression. The control group’s structured home-based exercises, despite being unsupervised, may have contributed to the absence of significant intergroup differences, emphasizing the need for future studies to compare varying supervision levels. Furthermore, technical challenges, such as internet connectivity issues and familiarity with video-based interventions, were not systematically assessed and may have influenced engagement and adherence. Another key limitation is the lack of standardized TR protocols, as variability in the session frequency, duration, and intensity could impact the outcomes. Future research should focus on multicenter trials with larger, more diverse cohorts, extended follow-ups, and the integration of standardized digital platforms to increase accessibility, engagement, and real-time monitoring. The inclusion of wearable technology and automated feedback systems may further improve adherence and optimize rehabilitation outcomes for MS patients.

## 5. Conclusions

This study highlights the efficacy of TR incorporating respiratory exercises in MS patients, which results in significant improvements in pulmonary function, fatigue, and quality of life. While no significant differences were detected between the TR and control groups, the TR approach presented unique advantages, including greater flexibility and accessibility, making it a viable alternative to conventional in-person rehabilitation. Future research should explore the application of TR in advanced-stage MS patients, assess its long-term efficacy, and evaluate emerging technologies such as wearable sensors and telemonitoring for real-time feedback.

## Figures and Tables

**Table 1:** Comparison of demographic and clinical characteristics between the telerehabilitation and control groups.

Variables	Telerehabilitation Group (*n* = 26)	Control Group (*n* = 26)	Test Value	*p*-Value
Gender				
Women	18 (69.2%)	17 (65.4%)	χ^2^ = 0.087	1.000
Men	8 (30.8%)	9 (34.6%)		
Smoking Status				
Non-smoker	18 (69.2%)	11 (42.3%)	χ^2^ = 4.182	0.117
Smoker	7 (26.9%)	11 (42.3%)		
Former smoker	1 (3.8%)	4 (15.4%)		
Age (years) $\bar{X}$ ± SD	33.08 ± 9.38	33.85 ± 9.36	t = 0.296	0.768
BMI (kg/m^2^) $\bar{X}$ ± SD	25.57 ± 4.97	25.81 ± 4.06	t = 0.188	0.851
EDSS Score $\bar{X}$ ± SD	1.98 ± 1.69	2.31 ± 1.35	t = 0.773	0.443

Values are presented as means ± SDs or numbers (%). Comparisons between groups were performed using the independent samples *t*-test for continuous variables and Chi-square test (χ^2^) for categorical variables. A *p*-value < 0.05 was considered statistically significant. Abbreviations: BMI—body mass index; EDSS—Expanded Disability Status Scale; $\bar{X}$—mean; ± SD—standard deviation; χ^2^—Chi-square test; t—independent samples *t*-test.

**Table 2:** Pulmonary function outcomes in the telerehabilitation and control groups.

	Telerehabilitation Group (*n* = 24)	Control Group (*n* = 24)	Inter-Group
	Mean ± SD	Mean ± SD	t (*p*)
FVC (L)			
Before	3.95 ± 0.96	3.99 ± 1.23	−0.12 (0.903)
After	4.01 ± 0.89	3.97 ± 1.21	0.14 (0.888)
Intra-group t (*p*)	−0.99 (0.330)	0.33 (0.745)	
FVC (% Predicted)			
Before	101.62 ± 12.70	95.96 ± 18.58	1.28 (0.206)
After	103.65 ± 11.42	97.31 ± 17.34	1.56 (0.125)
Intra-group t (*p*)	−1.17 (0.255)	−1.00 (0.326)	
FEV1 (L)			
Before	3.17 ± 0.83	3.25 ± 0.95	−0.32 (0.750)
After	3.39 ± 0.76	3.36 ± 1.03	0.14 (0.889)
Intra-group t (*p*)	−3.61 (0.001 *)	−2.24 (0.034 *)	
FEV1 (% Predicted)			
Before	94.81 ± 15.75	93.04 ± 15.69	0.41 (0.687)
After	97.54 ± 15.62	94.04 ± 14.21	0.85 (0.402)
Intra-group t (*p*)	−3.57 (0.001 *)	−1.30 (0.206)	
PEF (L)			
Before	5.32 ± 0.96	5.80 ± 2.20	−1.02 (0.314)
After	5.58 ± 0.95	5.93 ± 2.21	−0.74 (0.464)
Intra-group t (*p*)	−5.18 (0.000 *)	−3.27 (0.003 *)	
PEF (% Predicted)			
Before	73.43 ± 16.52	73.51 ± 19.84	−0.15 (0.988)
After	76.54 ± 15.10	76.12 ± 17.35	0.10 (0.925)
Intra-group t (*p*)	−4.74 (0.000 *)	−2.78 (0.010 *)	

Values are presented as the means ± SDs. Inter-group comparisons were performed using the independent samples *t*-test. Intra-group (pre-post) comparisons were performed using the paired samples *t*-test. * indicates statistically significant differences (*p* < 0.05). Abbreviations: FVC = forced vital capacity; FEV1 = forced expiratory volume in 1 s; PEF = peak expiratory flow.

**Table 3:** Comparison of functional and clinical outcomes in the telerehabilitation and control groups.

Variable	Telerehabilitation Group (*n* = 24)	Control Group (*n* = 24)	Intergroup (t, *p*)
FSS Scores			
Before	3.67 ± 1.80	3.95 ± 1.66	−0.59 (0.561)
After	3.23 ± 1.58	3.64 ± 1.57	−1.73 (0.91)
Intra-group (t, *p*)	2.45 (0.022 *)	−2.25 (0.045 *)	
MSQL-54			
PCS Before	56.42 ± 21.28	52.74 ± 21.10	0.63 (0.534)
PCS After	63.60 ± 19.66	57.13 ± 19.99	1.18 (0.245)
Intra-group (t, *p*)	−5.46 (0.000 *)	−4.23 (0.000 *)	
MCS Before	57.83 ± 19.97	52.14 ± 24.49	0.92 (0.363)
MCS After	62.41 ± 19.93	56.73 ± 22.72	0.96 (0.342)
Intra-group (t, *p*)	−2.21 (0.036 *)	−3.59 (0.001 *)	
6MWT			
Heart Rate Pre-test			
Before	87.00 ± 12.24	83.15 ± 11.17	1.18 (0.242)
After	85.15 ± 6.93	81.69 ± 6.83	1.81 (0.076)
Intra-group (t, *p*)	0.89 (0.001 *)	0.76 (0.455)	
Heart Rate Post-test			
Before	95.77 ± 12.97	93.23 ± 11.65	0.74 (0.461)
After	86.96 ± 7.15	84.50 ± 6.73	1.28 (0.207)
Intra-group (t, *p*)	3.90 (0.001 *)	3.77 (0.001 *)	
6MWT Distance (m)			
Before	317.69 ± 78.08	338.46 ± 67.18	−1.03 (0.309)
After	330.96 ± 74.32	351.92 ± 58.84	−1.13 (0.265)
Intra-group (t, *p*)	−2.75 (0.011 *)	−2.58 (0.016 *)	
Change in Heart Rate			
Before	8.77 ± 7.35	10.08 ± 10.46	−0.52 (0.604)
After	1.81 ± 2.91	3.00 ± 5.03	−1.05 (0.302)
Intra-group (t, *p*)	5.08 (0.000 *)	3.67 (0.001 *)	
Distance (m)			
Before	317.69 ± 78.08	338.46 ± 67.18	−1.03 (0.309)
After	330.96 ± 74.32	351.92 ± 58.84	−1.13 (0.265)
Intra-group (t, *p*)	−2.75 (0.011 *)	2.58 (0.016 *)	
Saturation Pre-test (%)			
Before	Median: 98 (95–99)	Median: 98 (95–99)	333.0 (0.921)
After	Median: 98 (85–99)	Median: 98 (85–99)	314.5 (0.652)
Intra-group (z, *p*)	0.00 (1.00)	1.10 (0.270)	
Saturation Post-test (%)			
Before	Median: 98 (97–99)	Median: 98 (82–101)	335.0 (0.953)
After	Median: 98 (92–99)	Median: 98 (92–99)	325.0 (0.803)
Intra-group (z, *p*)	−1.90 (0.058)	−0.14 (0.888)	

Values are presented as the means ± SDs or medians (min–max). Inter-group comparisons were performed using the independent samples *t*-test or Mann–Whitney U test, and intra-group (pre-post) comparisons were performed using the paired samples *t*-test or Wilcoxon signed-rank test based on data normality. * indicates statistically significant differences (*p* < 0.05). Abbreviations: FSS = Fatigue Severity Scale; MSQL-54 = Multiple Sclerosis Quality of Life-54; PCS = physical component summary; MCS = mental component summary; 6MWT = six-minute walk test.

## Data Availability

The raw data supporting the conclusions of this article will be made available by the authors on request.

## Supplementary Materials

The following supporting information can be downloaded at: https://www.mdpi.com/article/10.3390/medicina61040651/s1, Table S1: Comparison of respiratory function parameters between telerehabilitation and control groups; Table S2: Comparison of functional scores, quality of life, and exercise parameters between telerehabilitation and control groups.
