# Supplementary material for: Effects of Telehealth-Supervised Respiratory Exercise Training on Respiratory Function, Fatigue, Quality of Life, and Functional Capacity of Patients with Multiple Sclerosis
Source: Medicina (Kaunas). 2025 Apr 2;61(4):651. doi: 10.3390/medicina61040651 (PMC12028881; doi:10.3390/medicina61040651)
Supplement: Supplementary file 1 [file medicina-61-00651-s001.zip › medicina-3516240-supplementary.pdf]

Supplementary table S1. Comparison of respiratory function parameters between telerehabilitation and control groups.

Variables	Telerehabilitation Group (n=26)	Control Group (n=26)	Test Value	p-Value
**FVC (L)**	$\bar{X}$ = 3.93 ± 0.98	$\bar{X}$ = 3.99 ± 1.23	t = -0.123	0.903
**FVC (% Predicted)**	$\bar{X}$ = 101.88 ± 12.89	$\bar{X}$ = 95.96 ± 18.58	t = 1.281	0.206
**FEV1 (L)**	$\bar{X}$ = 3.17 ± 0.83	$\bar{X}$ = 3.25 ± 0.95	t = -0.320	0.750
**FEV1 (% Predicted)**	$\bar{X}$ = 94.81 ± 15.75	$\bar{X}$ = 93.04 ± 15.69	t = 0.406	0.687
**PEF (L)**	$\bar{X}$ = 5.32 ± 0.96	$\bar{X}$ = 5.80 ± 2.20	t = -1.016	0.314
**PEF (% Predicted)**	$\bar{X}$ = 73.43 ± 16.52	$\bar{X}$ = 73.51 ± 19.84	t = -0.150	0.988
**FEF %25-75 (L)**	$\bar{X}$ = 3.25 ± 1.35	$\bar{X}$ = 3.50 ± 1.22	t = -0.707	0.483
**FEF %25-75 (% Predicted)**	$\bar{X}$ = 74.39 ± 21.93	$\bar{X}$ = 79.81 ± 26.77	t = -0.798	0.428
**FEV1/FVC**	Median = 81.8 (78-97)	Median = 82.8 (79-100)	z = 263.50	0.168

Abbreviations: FVC: Forced Vital Capacity; FEV1: Forced Expiratory Volume in 1 Second; PEF: Peak Expiratory Flow; FEF %25-75: Forced Expiratory Flow at 25–75% of Pulmonary Volume; X̄: Mean; ±SS: Standard Deviation; Min: Minimum; Max: Maximum; t: Independent Samples t-Test; z: Mann-Whitney U Test.

Supplementary table S2. Comparison of functional scores, quality of life, and exercise parameters between telerehabilitation and control groups.

Variables	Telerehabilitation Group (n=26)	Control Group (n=26)	Test Value	p-Value
**FSS Scores**	$\bar{X}$ = 3.67 ± 1.80	$\bar{X}$ = 3.95 ± 1.66	t = -0.586	0.561
**MSQL-54**				
Physical Component Score (PCS)	$\bar{X}$ = 56.42 ± 21.28	$\bar{X}$ = 52.74 ± 21.10	t = 0.626	0.534
Mental Component Score (MCS)	$\bar{X}$ = 57.83 ± 19.97	$\bar{X}$ = 52.14 ± 24.49	t = 0.917	0.363
**6-Minute Walk Test (6MWT)**				
Heart Rate (Pre-test)	$\bar{X}$ = 87.00 ± 12.24	$\bar{X}$ = 83.15 ± 11.17	t = 1.183	0.242
Heart Rate (Post-test)	$\bar{X}$ = 95.77 ± 12.97	$\bar{X}$ = 93.23 ± 11.65	t = 0.742	0.461
Change in Heart Rate	$\bar{X}$ = 8.77 ± 7.35	$\bar{X}$ = 10.08 ± 10.46	t = -0.522	0.604
Distance (meters)	$\bar{X}$ = 317.69 ± 78.08	$\bar{X}$ = 338.46 ± 67.18	t = -1.028	0.309
**Saturation (%)**				
Pre-test	Median = 98 (95-99)	Median = 98 (95-99)	z = 333.0	0.921
Post-test	Median = 98 (97-99)	Median = 98 (82-101)	z = 335.0	0.953

Abbreviations: FSS: Fatigue Severity Scale; MSQL-54: Multiple Sclerosis Quality of Life-54; PCS: Physical Component Score; MCS: Mental Component Score; 6MWT: 6-Minute Walk Test; $\bar{X}$: Mean; ±SS: Standard Deviation; Min: Minimum; Max: Maximum; t: Independent Samples t-Test; z: Mann-Whitney U Test.
